# A Meta-Analysis of the Diagnostic Accuracy of Two Commercial NS1 Antigen ELISA Tests for Early Dengue Virus Detection

**DOI:** 10.1371/journal.pone.0094655

**Published:** 2014-04-11

**Authors:** Vivaldo G. da Costa, Ariany C. Marques-Silva, Marcos L. Moreli

**Affiliations:** Virology Laboratory, Federal University of Goiás, Jataí, Brazil; Icahn School of Medicine at Mount Sinai, United States of America

## Abstract

**Background:**

Dengue virus (DENV) NS1 antigen detection is regarded as an early diagnostic marker. Accordingly, several studies have evaluated the performance of tests that utilize NS1 capture, but the results of individual studies may be limited due to the restricted sample size of the patients recruited. Therefore, our objective was to perform a meta-analysis of the diagnostic accuracy of two commercial NS1 ELISAs (Panbio and Platelia).

**Methods and Results:**

Studies of interest were found in PubMed, Embase and Google Scholar databases using defined inclusion/exclusion criteria. A total of 30 studies containing 12,105 total enrolled patients were included. The results were as follows: 1) Panbio assays showed low overall performance, sensitivity 66% (95% confidence interval (CI) 61–71), specificity 99% (95% CI 96–100), positive likelihood ratio (LR+) 98 (95% CI 20–464), negative likelihood ratio (LR-) 0.3 (95% CI 0.2–0.4), diagnostic odds ratio (DOR) 289 (95% CI 59–1412); 2) Platelia assays showed high overall performance, sensitivity 74% (95% CI 63–82), specificity 99% (95% CI 97–100), LR+ 175 (95% CI 28–1099), LR- 0.3 (95% CI 0.2–0.4), DOR 663 (95% CI 98–4478). The lowest sensitivity values were for secondary infections (57% [95% CI 47–67] and 66% [95% CI 53–77] for Panbio and Platelia, respectively) and for the detection of DENV4. Regarding clinical manifestations, the sensitivity of Platelia was 69% (95% CI 43–86) and 60% (95% CI 48–70) for fever and dengue hemorrhagic fever, respectively. In addition, the sensitivity of both tests was slightly lower for samples from Southeast Asia and Oceania.

**Conclusion:**

DENV1 samples gave higher sensitivity results for both tests. We observed that factors negatively influencing the tests, such as the type of infection, geographical origins of samples and viral serotypes, require further investigation to optimize the diagnostic accuracy.

## Introduction

Dengue is a pandemic disease that has been neglected but is reemerging, putting approximately three billion people in tropical and subtropical regions at risk of this viral infection [1], [2]. Therefore, dengue poses a major threat to the public health systems of many countries, considering the occurrence of millions of cases and thousands of deaths annually [3].

Dengue virus (DENV), genus *Flavivirus*, is antigenically classified into four serotypes (DENV1-4). DENV is an arbovirus (Arthropod-borne virus) and is increasingly infecting humans, with the incidence of dengue showing a 30-fold increase within the last 50 years [3]–[6]. Dengue disease results in a wide clinical spectrum with undifferentiated febrile symptoms, hindering early diagnosis and clinical management. Thus, DENV infections can be asymptomatic or present as the classical clinical picture of dengue fever (DF). In revised WHO classification system (2009), DF was divided into dengue with or without warning signs and severe dengue. We will use the classification into DF/dengue hemorrhagic fever (DHF)/or dengue shock syndrome (DSS), since it continues to be widely used [2], [7].

Laboratory techniques involved in the diagnosis of DENV are based on the detection of viral genetic material, the specific detection of IgG/IgM antibodies and the detection of viral antigens, such as nonstructural protein 1 (NS1) [8]–[10]. NS1 is a glycoprotein that is abundantly produced by viruses in the early stages of infection, and it is found within the infected cells, in the cell membranes and secreted into the extracellular spaces [11], [12]. Therefore, one advantage of laboratory methods that perform NS1 antigen capture is the precocity of this marker, present at the onset of symptoms, in contrast to IgM, which is detected later, beginning at the fifth day of disease.

Currently, several laboratory methods that use the capture of DENV NS1 antigen are available [13]–[17]. The successful implementation of these methods reflects on the good performance of these tests. Despite the existence of several studies evaluating tests for an NS1 capture ELISA assay, no meta-analysis evaluating the diagnostic accuracy of these commercial kits has been performed. Due to the limited sample size of patients recruited in individual studies, meta-analysis may increase the accuracy of estimates of individual studies. Therefore, we conducted a meta-analysis of the accuracy of diagnosis for Panbio NS1 and Platelia NS1 ELISA assay kits to obtain the overall estimated and summarized performance of the tests in the detection of DENV.

## Materials and Methods

### Search strategy

This meta-analysis was guided by the standard PRISMA protocol (Preferred Reporting Items for Systematic reviews and Meta-analysis (Table S1)) and methods proposed by the Cochrane Collaboration [18], [19]. PubMed; Embase and Google Scholar databases were searched for articles using a combination of descriptors to select the studies of interest.

### Study selection

After finding previously published studies in the databases with the descriptors “dengue” OR “dengue virus” AND “diagnosis” OR “ELISA NS1” OR “early diagnostic” OR “diagnostic accuracy” OR “performance test”, we performed an analysis on the inclusion/exclusion criteria.

As inclusion criteria, we used studies that evaluated the sensitivity and specificity parameters of ELISA kits involving the capture of dengue NS1 antigen and the Panbio (Alere, Brisbane, Australia) or Platelia (Marnes-la-Coquette, France; Hercules, CA, USA (Bio-Rad)) kits.

As exclusion criteria, we did not review studies that were not published in English, Spanish or Portuguese or studies with limited information for calculating sensitivity and specificity. We excluded specific articles types, for instance review articles, comments, the editorial, letters and conference abstract. Additionally, two authors reviewed the studies independently, in case of disagreement a third author was consulted.

### Data extraction

The following data from each study included in the meta-analysis were extracted: author, year of publication, place of study, gender, age and number of participants, method of diagnosis, study design, sensitivity and specificity data, positive predictive value (PPV) and negative predictive value (NPV). The data to be extracted were analyzed in the following subgroups: classification of infection, viral serotype, period of the collection of samples, geographic origin of patients and clinical picture presented. Subsequently, the data related to the accuracy of the diagnosis were plotted on a 2×2 contingency table.

### Quality assessment

The analysis of the quality of the studies was performed based on a tool known as the quality assessment of diagnostic accuracy studies (QUADAS), which allows for the identification of important design elements in diagnostic accuracy studies [20]. The QUADAS tool consists of 14 key items (sufficient test description and reference, representative spectrum, reported withdrawals and indeterminate results, relevant clinical information, index test results blinded, definition positive test result, cutoff values, complete verification of diagnosis, avoided clinical review bias, appropriate selection and reference standard, and acceptable detail between tests). Items are evaluated using a score of “low”, “high”, or “obscure”, which are formulated for an answer as “no”, “yes” and “unclear”, respectively, that may indicate low or high risk of publication bias. Each of 14 items was scored from 1 to 0, with a total quality score between 11 and 14 was considered “good”, between 7 and 10, “moderate”, and 6 or less was considered “poor”.

### Statistical analysis

STATA IC/64 software (version 13.1, College Station, TX) with MIDAS and METANDI commands was used for the meta-analysis. For correction in the cells containing zero values, correction factors from METANDI commands were used.

The sensitivity (true positive rate), specificity (true negative rate), positive likelihood ratio and negative (LR+, or LR-, is estimated by the ratio of the proportion of positive, or negative, tests in the diseased versus no-diseased subjects) and diagnostic odds ratio (DOR is calculated as the LR+ divided by the LR-), with a confidence interval (CI) of 95%, were obtained for each study and subsequently combined. Cochran Q chi-square test and the I^2^ statistic were explored to assess the heterogeneity of the included studies. Random-effects model was used if the result of the Q test was significant (p<0.05) and I^2^>50%. The meta-regression was planned to be used if there was high heterogeneity (I^2^>50%) [21]. Additionally, an hierarchical summary receiver operating characteristic (HSROC) type curve of the selected studies was then plotted with the software. The HSROC curve is a bivariate model that provides information on the overall performance of a test through different thresholds. We also constructed the summary receiver operating characteristics (SROC) curve and the respective area under the curve that serves a global measure of the test performance [22].

To assess potential publication bias, we used the Deeks funnel plot, with p<0.05 indicating the presence of publication bias [23]. Fagan nomograms, a two-dimensional graphical tool for estimating how much the result of a diagnostic test changes the probability that a patient has a disease, was also used to estimate the clinical value of the index test, which is based on the LR+ and LR- obtained from the meta-analysis [24].

## Results

**Figure 1:**
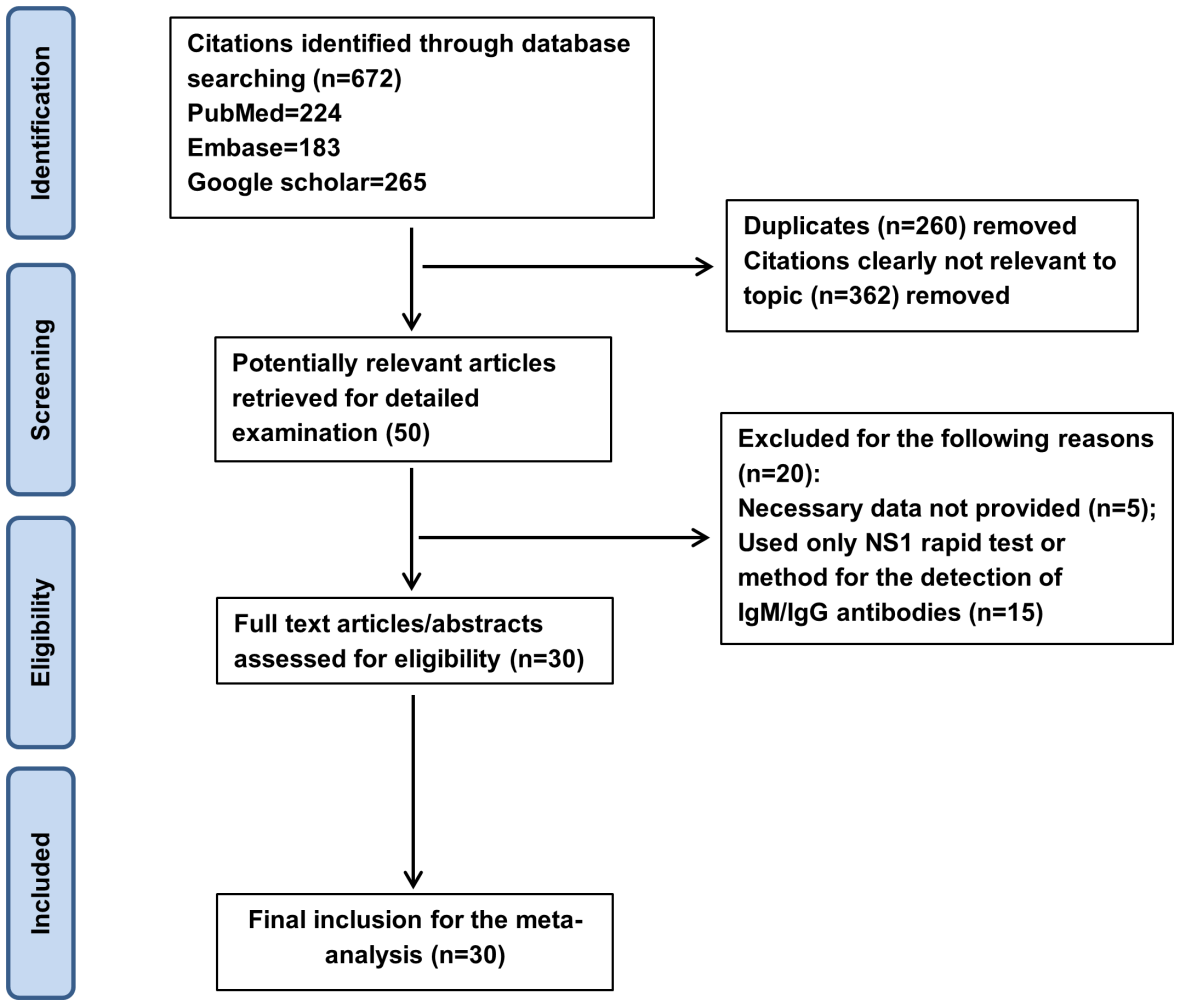
Flowchart of the steps performed in the meta-analysis.

Our search found 672 citations related to dengue through the combined application of descriptors in the three databases described above. During the final stage of selection, we excluded five studies that only assessed the sensitivity of laboratory methods or did the overlap the results of the two tests in their analysis [13]–[17]. After the exclusion criteria were applied, 30 baseline studies remained [25]–[54], which were included in our meta-analysis because they involved experimental research evaluating the diagnostic accuracy of the Panbio or Platelia kits, which used NS1 antigen capture in an indirect ELISA format. The results of our literature search are shown in Figure 1.

Among the studies included in the meta-analysis, there were a total of 12,105 patients recruited. These patient samples were collected from 17 countries in Latin America, Asia and Oceania, and the most studies were conducted in Brazil (27%) [29], [34], [37], [41], [45], [47], [52], [54], Vietnam (13%) [32], [33], [36], [49], Malaysia (13%) [26], [36], [42], [46] and Thailand (13%) [31], [36], [43], [48]. Regarding the design of the studies, they were classified into two types: prospective and retrospective cohorts, of which only twelve reported that their samples were collected during dengue outbreaks [25], [26], [30], [37], [39], [40], [44], [45], [51]–[54]. Typically, most samples were collected until the sixth day of the onset of symptoms [25], [27], [28], [30]–[37], [40]–[42], [47], [49], [50], [52]–[54]. The data extracted from the final selection are shown in Table 1.

**Table 1:** Summary of the included studies.

Ref.	Study Design	Location	Sample (n)	Sex	Median Age, y	Results^PANBIO^				Results^PLATELIA^				Dengue prevalence (%)	Diagnostic method
						Sen.% (95% CI)	Spec.% (95% CI)	PPV% (95% CI)	NPV% (95% CI)	Sen.% (95% CI)	Spec.% (95% CI)	PPV% (95% CI)	NPV% (95% CI)		
25	Cohort, prospective	French Guiana	349	Female 75%	33					88.7 (84–92.4)	100 (84.9–100)	89.8	100	72	ELISA; RT-PCR; VI
26	Cohort, retrospective	Malaysia	354	NR	NR					93.4	100	100	98.9	37.5	ELISA; RT-PCR; VI
27	NR	Puerto Rico	253	NR	NR	64.9 (58.2–71.1)	97.8 (88.4–99.6)	100 (97.2–100)	39.3 (30.7–48.5)	83.2 (77.5–87.7)	100 (92.1–100)	100 (97.2–100)	62.5 (51–72.8)	82	ELISA; Real time RT-PCR; VI
28	Cohort, prospective	Laos	92	NR	NR	63.2 (53.4–73)	100	100	79.4 (71.2–87.7)					41	ELISA; RT-PCR
29	Cohort, prospective	Brazil	250	Male 58%	35					85.4	94	81	95.6	32	ELISA; Real time RT-PCR; VI
30	Cohort, retrospective	French Guiana	320	NR	NR	55.1 (49–61.2)	97.9 (88.9–99.9)	75.2	98	82.4 (77.3–86.7)	100 (92.6–100)	100	100	69	ELISA; ICG; RT-PCR; VI
31	Cohort, prospective	Thailand	235	Male 56.2%	17.8					63.2 (55.7–70)	98.4 (91.7–99.7)	99	52.5	72.8	ELISA; RT-PCR; VI
32	Cohort, prospective	Vietnam	138	Male 44.9%	16					83.2 (75.5–89.3)	100 (86.7–100)	100 (97.2–100)	38.2 (22.2–56.4)	90	ELISA; RT-PCR
33	NR	Vietnam	459	Male 55%	18.3					37	99.5	90.9	92.2	12	ELISA; RT-PCR
34	Cohort, prospective	Brazil	92	NR	NR					70 (59–79.2)	100 (54.1–100)	100 (94–100)	18.7 (7.2–36.4)	93	ELISA; Real time RT-PCR
35	NR	Venezuela	147	NR	NR	60.9 (50.4–70.5)	94.4 (80.9–99.4)	100	41.4	71.3 (61–80)	86.1 (70.9–94.4)	100	49	59	ELISA; ICG; RT-PCR; VI
36	Cohort, prospective	M	2259	NR	NR	52	90	76.2	90	66	100	82.3	100	76	ELISA; RT-PCR; VI
37	Cohort, retrospective	Brazil	450	NR	NR	72	100	100	78	84	99	98	86	49	ELISA; RT-PCR; VI
38	Cohort, retrospective	Colombia	310	NR	NR	71.1 (64.6–77)	89.1 (80.9–94.7)	94 (89.1–97.1)	56.6 (48.1–64.8)	70.8 (64.1–76.8)	92.3 (84.8–96.9)	95.5 (91–98.2)	57.5 (49.1–65.7)	70	ELISA; RT-PCR; VI
39	Cohort, prospective	Singapore	433	NR	NR	67 (57.3–75.7)	100 (96.4–100)	100 (96.4–100)	73.5 (64.3–81.4)	81.7 (73.1–88.4)	100 (96.4–100)	100 (96.4–100)	83.3 (75.3–88.2)	37	ELISA; ICG; RT-PCR
40	Cohort, prospective	India	2070	NR	NR	61.4	100	100	100					41	ELISA; RT-PCR;
41	Cohort, prospective	Brazil	86	Female 62.5%	27	50 (29.9–70.1)	100 (94–100)	66	84.8					30	ELISA; RT-PCR; VI
42	Cohort, prospective	Malaysia; China; India	558	Male 62%	26					91.6	100	92.3	95.8	34	ELISA; Real time RT- PCR; VI
43	Cohort, prospective	Thailand	85	NR	NR					76.4	100 (82.8–100)	100	62	65	ELISA; ICG; VI
44	Cohort, prospective	Cambodia	339	Female 52.3%	4					57.7 (51.4–63.8)	100	100	41.8 (34.7–49.2)	72	ELISA; RT-PCR; VI
45	Cohort, retrospective	Brazil	450	NR	NR	80	100	100	100					51	ELISA; RT-PCR; VI
46	NR	Malaysia	208	NR	NR					83.7	90.4	86	95	38	ELISA; RT-PCR
47	Cohort, prospective	Brazil	147	NR	NR	87.5	71	68	85	95	47	58	92	47	ELISA; ICG; HI
48	NR	Thailand-Myanmar	162	Male 60.5%	23	54.6 (42–66)	100 (96–100)	100 (91–100)	73.2 (64.4–80.8)					44	ELISA; Real time RT-PCR
49	Cohort, prospective	Vietnam	116	NR	NR					64.7 (54.5–74.9)	95.8 (87.8–100)	73.9	96	82	ELISA; RT-PCR
50	Cohort, retrospective	Thailand	626	NR	NR	44.8 (38–51)	93.2 (88–97)	87.5	92.5	56.5 (50–63)	100 (98–100)	84	100	71	ELISA; RT-PCR; HI
51	Cohort, prospective	Indonesia	503	NR	NR	56.4	100	100	43					42	ELISA; RT-PCR; VI
52	Cohort, prospective	Brazil	220	NR	NR	82	91	85	89					31	ELISA; RT-PCR
53	Cohort, retrospective	Indonesia	275	Male 68%	30.7					46.8 (40.2–53.3)	100	100	32	80	ELISA; RT-PCR;
54	Cohort, prospective	Brazil	119	NR	NR					0	100	0	51	49	ELISA; RT-PCR; VI

Abbreviations: Ref, Reference studies; y, year; Sen., Sensitivity; Spec., Specificity; NR, Not reported; M, Multicenter; HI, Hemagglutination inhibition; ICG, Immunochromatographic; VI, Virus isolation.

**Figure 2:**
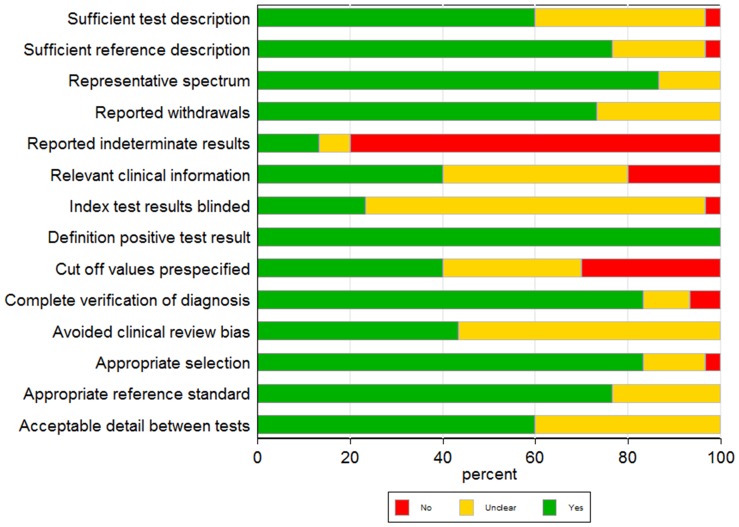
The assessment of methodological quality items shown for all included studies. Proportions of studies rated as “yes”, “no”, or “unclear” for each QUADAS item.

The QUADAS tool consists of 14 items, and the results of the analysis can be seen in Figure 2. The quality of all studies was generally moderate, with median QUADAS score of 9 (Table S2). However, the items related to the determination of the indeterminate results, including relevant clinical information (classification DF or DHF) and disclosure of the cut-off and blinding of samples before processing by laboratory tests, were evaluated items that presented more risk of bias. Additionally, the Deeks funnel plot did not show potential publication bias for the two subgroups of studies (p = 0.56 and p = 0.09) (Figure S1), yet a significant amount of heterogeneity were detected for the two tests (I^2^ ranged from 85% to 97%). Meta-regression showed that the covariates, origin of the samples, period of sample collection and retrospective *versus* prospective samples were items that contributed to diversity among studies (Table S3).

### Overall accuracy of the Panbio and Platelia commercial kits

**Figure 3:**
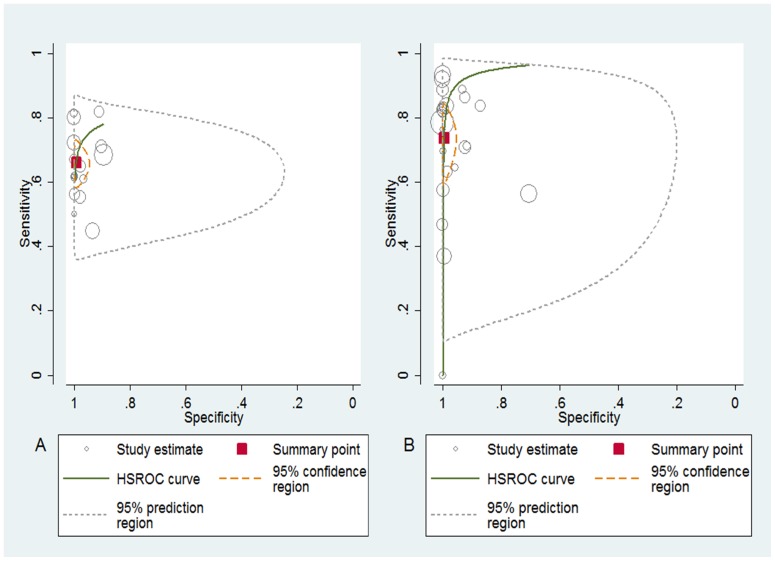
HSROC plot displaying diagnostic accuracy results of included studies. Panbio (A) and Platelia (B) kits. The circle diameter (study estimate) is proportional to the weight given to each study. Summary sensitivity and specificity is marked by a red square.

Among the selected studies, 16 assessed the trials of Panbio [27], [28], [30], [35]–[41], [45], [47], [48], [50]–[52] and 23 assessed the trials of Platelia [25]–[27], [29]–[39], [42]–[44], [46], [47], [49], [50], [53], [54]. In relation to Panbio, the sensitivity, specificity, LR+, LR- and DOR overall were 66% (95% CI 61–71), 99% (95% CI 96–100), 98 (95% CI 19–367), 0.3 (95% CI 0.2–0.4) and 289 (95% CI 59–1412), respectively. Similarly, the values for Platelia were 74% (95% CI 63–82), 99% (95% CI 97–100), 175 (95% CI 28–1099), 0.3 (95% CI 0.2–0.4) and 663 (95% CI 98–4478), respectively. The area under summary ROC curve were 0.84 (95% CI 0.80–0.87 (Panbio)) and 0.96 (95% CI 0.94–0.97 (Platelia)) (Figure S2) and the graphs of the HSROC curves of the individual studies for the diagnostic accuracy of two tests analyzed are shown in Figure 3A–B.

### Accuracy of the tests on the viral serotype and classification of infection

When evaluating the accuracy of tests for sensitivity, DOR, LR+ and LR- for serotype DENV1, we obtained the following values: 81% (95% CI 73–87), 702 (95% CI 101–4842), 136 (95% CI 23–806) and 0.2 (95% CI 0.1–0.3), respectively, for Panbio. Similarly, the values for Platelia were 90% (95% CI 81–95), 5460 (95% CI 131-225878), 526 (95% CI 12-21602) and 0.09 (95% CI 0.04–0.19).

For DENV2, pooled sensitivity was 74% (95% CI 67–80), DOR was 507 (95% CI 55–4663), LR+ and LR- were 133 (95% CI 17-1023) and 0.3 (95% CI 0.2–0.4), respectively, for Panbio. In Platelia, pooled sensitivity was 73.3% (95% CI 61–83), DOR was 714 (95% CI 37-13466), LR+ and LR- were 191 (95% CI 12-3090) and 0.26 (95% CI 0.17–0.4), respectively.

For DENV3, pooled sensitivity was 70.7% (95% CI 63–78), DOR was 481 (95% CI 33-6869), LR+ and LR- were 141 (95% CI 11-1780) and 0.3 (95% CI 0.2–0.4), respectively, for Panbio. In Platelia, pooled sensitivity was 83% (95% CI 75–89), DOR was 2353 (95% CI 72-7e+4), LR+ and LR- were 397 (95% CI 12-13119) and 0.16 (95% CI 0.11–0.25), respectively.

For DENV4, pooled sensitivity was 37% (95% CI 26–50), DOR was 18 (95% CI 6–63), LR+ and LR- were 12 (95% CI 4–38) and 0.6 (95% CI 0.5–0.8), respectively, for Panbio. In Platelia, pooled sensitivity was 58% (95% CI 30–81), DOR was 96848 (95% CI 14–6e+8), LR+ and LR- were 41006 (95% CI 6–3e+8) and 0.4 (95% CI 0.2–0.8), respectively.

Regarding the classification of dengue primary or secondary infection types, the following global estimates for primary infection were obtained for the parameters of sensitivity, DOR, LR+ and LR-: 75% (95% CI 66–82.5), 7114 (95% CI 18–2e+6), 1761 (95% CI 5-601666) and 0.24 (95% CI 0.17–0.34), respectively, for Panbio, and 94.6% (95% CI 91–97), 2036 (95% CI 341–12130), 110 (95% CI 23–518) and 0.05 (95% CI 0.03–0.09), respectively, for Platelia. For secondary infection, these laboratory indices were 57% (95% CI 47–67), 3443 (95% CI 12–9e+5), 1484 (95% CI 6–4e+5) and 0.4 (95% CI 0.3–0.5), respectively, for Panbio, and 66% (95% CI 53–77), 632 (95% CI 47–8374), 216 (95% CI 13–3453) and 0.3 (95% CI 0.2–0.5), respectively, for Platelia.

### Accuracy of the tests regarding clinical manifestations of dengue

**Figure 4:**
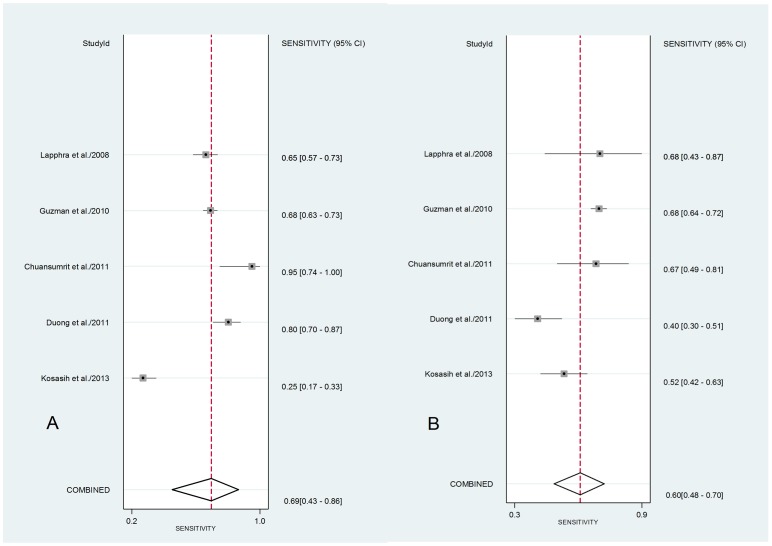
Forest plot of the sensitivity of Platelia kit. Forest plot of the sensitivity of each study and pooled sensitivity for studies that distinguished clinical features of patients infected with DENV into DF (A) and DHF (B). The sensitivity is represented by the circles in squares and the horizontal lines represent the point estimate (95% CI for each included study). Diamonds represent the pooled estimate (95% CI).

To verify whether patients with moderate clinical forms (DF) or severe dengue (DHF/DSS) showed significant variations in the performance of the tests, we performed a global estimate of the accuracy of the tests. In this case, only Platelia was used for laboratory evaluation of the different clinical forms of the patients. Only five studies performed this calculation, with forest plot of sensitivity showing values that ranged from 25% to 95% (Figure 4A–B). The pooled sensitivity was 69% (95% CI 43–86) and 60% (95% CI 48–70) for DF (A) and DHF (B), respectively.

### DOR and post-test probability

The DOR is commonly considered a global measure of test performance that summarizes the diagnostic accuracy of the index test as a single number that describes how many times greater the chance is of getting a positive result in a person with the disease than in someone without the disease. As described above, the values of DOR were considerably high due to the high values of sensitivity and principally of the specificity observed in this study. In this case, a function of DOR plotted on the graph would present an exponential behavior, rising abruptly and presenting a clear positive correlation with the sensitivity and specificity [55], [56].

**Figure 5:**
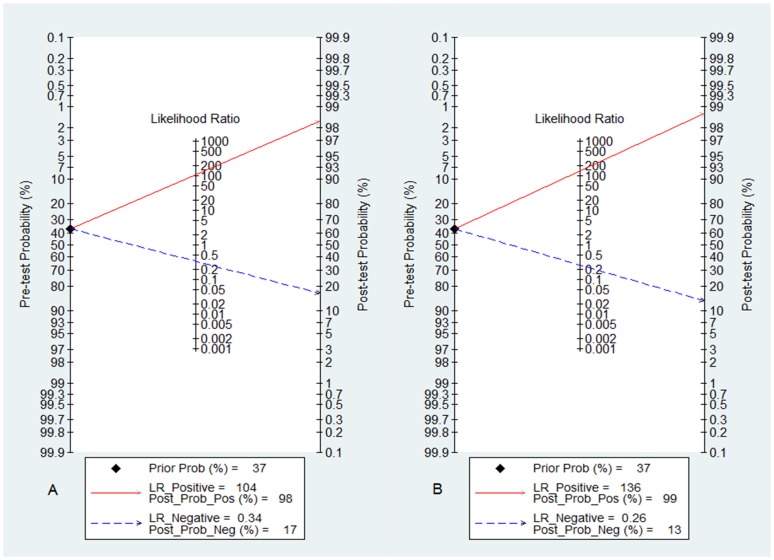
Fagan's nomogram for the calculation of post-test probabilities. A pre-test probability of 37% for dengue disease was fixed, which was estimated by the number of symptomatic cases in selected studies. (A) Panbio had a post-test probability of 98%. For Platelia kits (B) post-test probability was 99%, ie, with an estimated prevalence of 37%, if this patient tests positive, the post-test probability that she truly has dengue would be 99% (solid line in red). On the other hand, if patient tests negative, the post-test probability that she truly has dengue would be 17% (A) or 13% (B) (blue dotted line). The results were obtained by the following calculations: pretest odds = prevalence/1-prevalence; post-test odds = pretest odds x LR- (LR+); post-test probability = post-test odds/1+post-test odds. LR, likelihood ratio.

To obtain the post-test probability, we used Fagan's nomogram for which we performed a simulation of an environment that had a prevalence of 37% for dengue disease, with base on the studies selected. Thus, the probability in this model of someone having the disease and not being detected by the NS1 Panbio ELISA test was 17%. In the same situation for the NS1 Platelia ELISA test, a negative result was associated with 13% of individuals with the disease (Figure 5A–B). In contrast, the post-test probability of sick patients with a positive test was 98% and 99%, respectively, for Panbio and Platelia. Thus, showing that these tests specifically capture the NS1 antigen is important in the diagnosis of dengue.

## Discussion

The studies included in this meta-analysis had a global sensitivity and specificity ranging from 45% to 100% and 71% to 100%, respectively, for Panbio and ranging from 0% to 95% and from 47% to 100%, respectively, for Platelia. When we performed an overall estimate of sensitivity, a superiority of Platelia (74% [95% CI 63–82]) over Panbio (66% [95% CI 61–71]) was detected. With respect to this increased sensitivity of the former test, there are no hypotheses explaining this outcome, but it has been observed that viral serotype can influence the accuracy of the test, thereby changing the sensitivity and resulting in both the Panbio and Platelia tests having higher sensitivity for DENV1. However, there were more participants with DENV1 included for analysis using the Platelia test [25], [27], [28], [30], [35]–[37], [39], [44], [47], [53], a fact that may have influenced the overall estimate, although our results are similar to the scientific literature, which demonstrates a higher sensitivity for Platelia.

Our findings in relation to the sensitivity of the tests against viral serotypes are partially consistent with the scientific literature, with a lower sensitivity observed for both tests (37% for Panbio and 58% for Platelia) for DENV4. However, some studies have also reported a lower sensitivity for Platelia for DENV2 [30], [35], [36], [47], [50]. The low accuracy of the Panbio during an epidemic of DENV4 and Platelia were recently analyzed by Colombo et al. [52] and Sea et al. [54], who observed the occurrence of false negatives. It is not yet known why the NS1 ELISA has a lower sensitivity in the patients infected with DENV4, but some hypotheses can be postulated: (i) there could be quantitative differences in the secreted NS1 form, depending on the viral serotype, which may lead to less availability of NS1 and reduced chances of detection, (ii) the higher incidence of DENV4 in secondary infections, and (iii) there could be presence of polymorphism in the NS1 gene associated with immune epitopes. However, these speculations require additional studies to confirm them.

*In silico* analysis using the Virus Pathogen Resource software (ViPR: www.viprbrc.org) to assess the degree of variability of the NS1 protein of DENV1-4 serotypes of the complete sequences from Asia (1466 strains) *versus* South America (476 strains) revealed the existence of significant variability between NS1 sequences. At least 83 amino acid positions were identified (Table S4). However, in the known consensus NS1 region “^111^LRYSWKTWGKA^121^” [57], there was only one polymorphism (replacing R with K at position 112) in DENV4; this polymorphism was found in 23 strains from South America and Asia. Thus, it appears that this polymorphism is not the main factor influencing the test but will be important for understanding whether external variant amino acids can have some influence on the immune epitope.

The detection and semi-quantitation of NS1 is proportional to the optical density (OD) measured at 450/620 nm [10]. The kits that were analysed determine the cut off by the average and standard deviations of the OD values from calibrators and the values are expressed in scales that can be interpreted as negative, indeterminate or positive. The calibration curve for the detection of NS1 is obtained by comparing different dilutions of the antigen, and the resulting values are expressed in OD units, as measured using an ELISA reader. Accordingly, Young et al. [12] calculated the linear portion of the standard curve to determine the serum NS1 concentration, obtaining a minimum threshold of 4 ng/ml. If there are quantitative differences in the secretion of NS1, depending on the different DENV serotypes, this could reduce the sensitivity of the test when certain dilutions are made. Indeed, if NS1 DENV4 is present in smaller amounts, it would be interesting to increase the detection test by obtaining a new calibration curve with lower dilutions of the test samples.

The determination of dengue diagnosis only with clinical and epidemiological data may result in errors [58], [59]. To circumvent this problem, a laboratory diagnosis is crucial for correct identification. One of the main benefits of laboratory methods is to allow the screening of patients suspected of such diseases to implement the most appropriate clinical management and to provide greater efficiency of the epidemiological surveillance system. The epidemiological surveillance systems are important in the control of outbreaks, as in cases of dengue, where movement of a new serotype of DENV often proceeds to epidemic proportions. The constant occurrence of dengue outbreaks can result in a higher incidence of secondary infections, which are positively correlated with a higher risk of DSS [60]. In this context, a lower sensitivity in patients with secondary infections was found for both analyzed tests, a fact that is worrisome because it would be useful to have a better test accuracy at this stage, due to the possibility of these patients progressing to more severe forms of the disease.

The “original antigenic sin” proposed by Sabin (1952) notes that, in successive infections by DENV, antibody memory may confer only transient protection against heterologous serotype infections, so that antibodies generated in secondary infections would be more effective in the neutralizing the viral serotype that caused the primary infection instead of the secondary one. The overall assessment of the sensitivity of both kits indicated that it was considered elevated in patients with primary infection (77% Panbio; 95.5% Platelia). In contrast, in secondary infections, there was a loss of sensitivity for both kits (24% for Panbio and 31% for Platelia). It is likely that this is due to the increased supply of antibodies, although weakly neutralizing [61], binding to the NS1 antigen. Accordingly, to increase the sensitivity of the tests, complex NS1 antibodies are separated by treatment of the test samples with acid [62]. Although this method has improved the sensitivity of the tests, only one study [31] among the ten that distinguished the types of infections [26], [28], [29], [32], [37], [39], [45], [51], [53] performed this step to dissociate antigen and antibody.

We conducted an overall estimate of the sensitivity of the kits correlated with the geographic origin of patients. The tests had a slight better accuracy for samples from Latin America, with values of 70% (95% CI 63–76 [Panbio]) and 80% (95% CI 75–85 [Platelia]), while these rates for patients who were from Southeast Asia and Oceania were 59% (95% CI 51–66 [Panbio]) and 73% (95% CI 61–82 [Platelia]). From these findings, it cannot be inferred that there were significant differences between geographical origins because even these results differ from the multicenter study conducted by Guzman et al. [36]. We believe that the differences in the sensitivity of the tests are most likely attributed to the process of the epidemiological evolution of DENV serotypes, in which a greater restriction of species from the same geographical region may be the result of the viral ancestral lineage [63]. This theory is supported by a phylogenetic analysis that elucidated the origins and molecular evolution of DENV in different geographic regions of world and showed the high genetic diversity of dengue, in which there are several clusters of different sublineages even within a single genotype [64]. In this context, Watanabe et al. [65], through studies in mice, have found that the secretion of NS1 is dependent on the viral strain. This reinforces the idea that co-circulating viral strains can affect the accuracy of tests that detect the NS1 antigen.

Although we mention above that the observed differences in laboratory parameters, in association with different geographical origins of patients, are mainly due to a process of molecular evolution of DENV, we cannot rule out the influence of the host in these molecular dynamics. In this sense, Mairiang et al. [66] found that there are several interactions between the protein of DENV and human and mosquito hosts. Therefore, there may be an interaction between the genotype of the host and the pathogen (DENV), although this has been demonstrated only in the main dengue vector, *Aedes aegypti* [67]. While the host may influence the molecular dynamics of the evolution of the pathogen, to what extent this influences the pathogen infecting humans in certain geographical regions and how this may affect the laboratory methods used should be defined to improve the performance of assays.

The DOR obtained in this meta-analysis was on average usually greater than 500. In fact, the high variations of values for sensitivity and specificity were also reflected in the DOR, which may vary from zero to infinity with higher values denote a better discriminatory diagnostic test [56]. Additionally, the post-test (Fagan's nomogram) probability was also high (Figure 5A–B), indicating a good clinical utility of the tests, although caution is needed in their interpretation because the samples included in the studies were mostly from symptomatic patients suspected of dengue, which increased the overall rates of prevalence.

In addition to the NS1 ELISA, there are immunochromatographic methods, which are known as rapid tests because results are obtained on average within 30 minutes. Several studies have evaluated the NS1 rapid tests, with sensitivities ranging from 51% to 90% [13]–[17], [38]. So although very good methods for identification of DENV infections exist and are in use, it is prudent to include additional methods whenever possible. We believe that one of the best choices is the combination of NS1 ELISA methods and an NS1 rapid test, along with a method for detection of IgG to increase sensitivity in secondary type infections.

Our meta-analysis had several limitations. First, there are two generations of the Panbio NS1 ELISA, and the latest second-generation kit had a higher sensitivity. Among the analyzed studies, few authors identified the generation of the kits used in their experiments, so our overall estimate of the sensitivity of Panbio could be influenced by this aspect. Second, although the specificity was almost 100%, it should be noted that only a few authors used a different pathological group of dengue, while most used samples from healthy individuals and blood donors. We understand that this happens because of the abundance and ease of obtaining these samples, but it is critical to avoid biases and to test the assays more in relation to other *flavivirus* and similar diseases. Third, only a third of the studies made a distinction between or disclosed the serotypes of DENV. Perhaps this factor, along with precocity of the samples, had a greater influence on the accuracy of tests. Fourth, there was statistically significant heterogeneity across the included studies. In an effort to explore source of heterogeneity, meta-regression revealed that origin, period and retrospective samples might is causing diversity on sensitivity and specificity. Fifth, only a few studies reported that the samples were from primary or secondary infections. Sixth, we used available data to calculate the sensitivity up to the sixth day of blood collection; however, some studies had a period of sample collection lasting until the ninth day of the febrile phase and this factor can also significantly compromise the accuracy of tests. Finally, data were not divided into additional groups based on other variables, such as gender or age, due to the limitations of original information for each patient included in the studies.

In conclusion, despite the above limitations mentioned, this meta-analysis showed a good overall estimate of sensitivity ranging from 66% (95% CI 61–71 [Panbio]) to 74% (95% CI 63–82 [Platelia]). Specificity was near 100% for both kits. The main factors influencing the diagnostic accuracy were the type of infection (primary *versus* secondary), viral serotype, geographical origins of samples and how early the samples were collected. However, to what extent and how these factors affect the diagnostic accuracy require more studies in order to optimize these tests.

## Supporting Information

Figure S1 **Deek's funnel plot asymmetry test for publication bias.** Deek's funnel plot asymmetry test not suggested potential publication bias (p = 0.56 in the Panbio kit (a)), (p = 0.09 in the Platelia kit (b)).
(TIF)

Figure S2 **Summary ROC curve plot with sensitivity and specificity for Panbio (A) and Platelia (B).** Each large X represents individual study in meta-analysis. Summary operating point is a single sensitivity/specificity point estimated by the results of studies. AUC =  area under the curve.
(TIF)

Table S1 **PRISMA Checklist.**
(DOC)

Table S2 **Methodological quality of the 30 included studies.**
(XLSX)

Table S3 **Univariate meta-regression analyses of the sensitivity and specificity.**
(DOCX)

Table S4 **Amino acid positions of NS1 identified with significant variations.** The positions and variability were obtained by the crossing of the NS1 strains DENV1-4 from Asia and South America.
(XLSX)
