# Supplementary figures and images for: A Meta-Analysis of the Diagnostic Accuracy of Two Commercial NS1 Antigen ELISA Tests for Early Dengue Virus Detection
Source: PLoS One. 2014 Apr 11;9(4):e94655. doi: 10.1371/journal.pone.0094655 (PMC3984211; doi:10.1371/journal.pone.0094655)

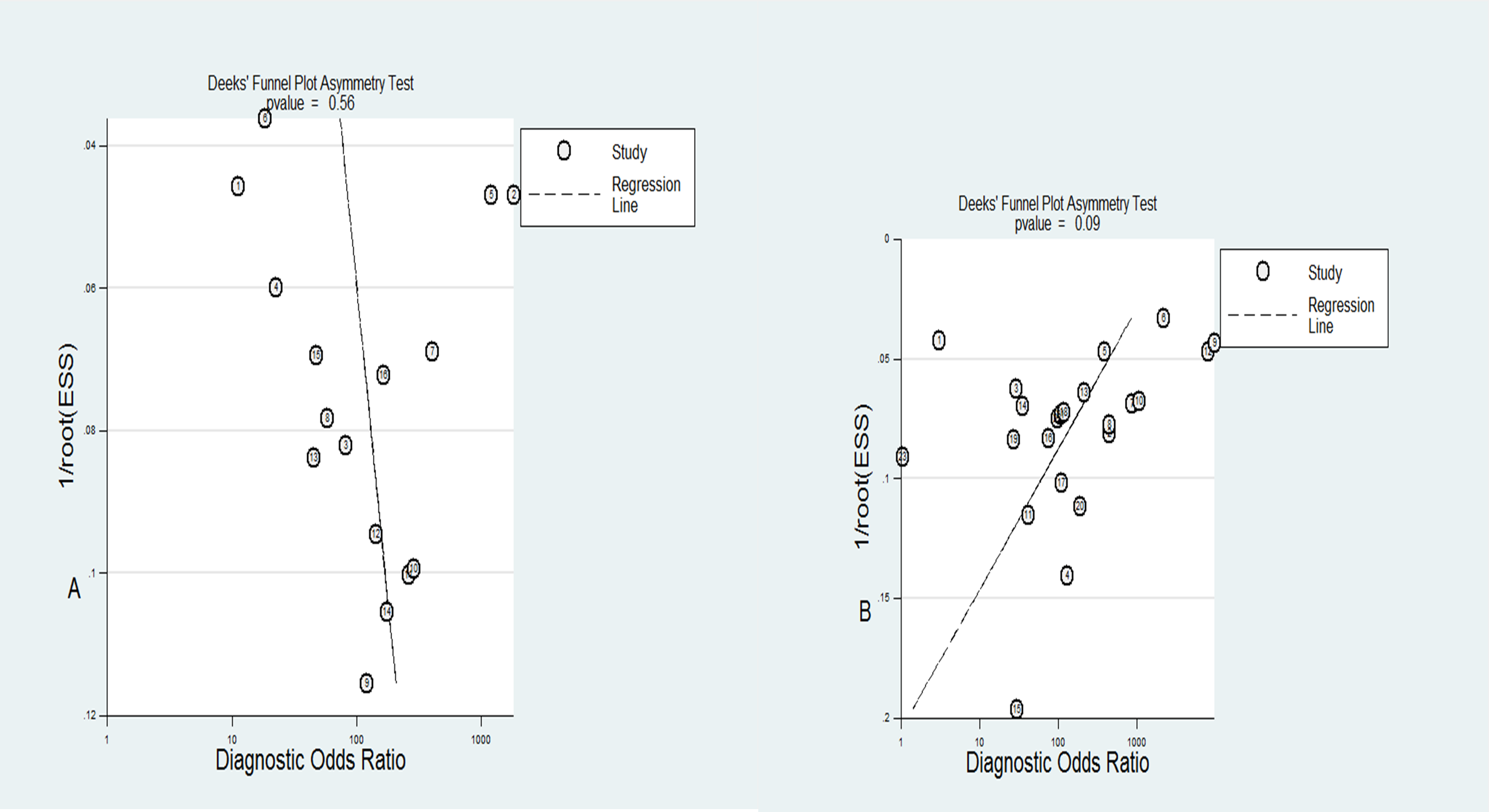

Supplement: Figure S1 — Deek's funnel plot asymmetry test for publication bias. Deek's funnel plot asymmetry test not suggested potential publication bias (p = 0.56 in the Panbio kit (a)), (p = 0.09 in the Platelia kit (b)). (TIF) [file pone.0094655.s001.tif]

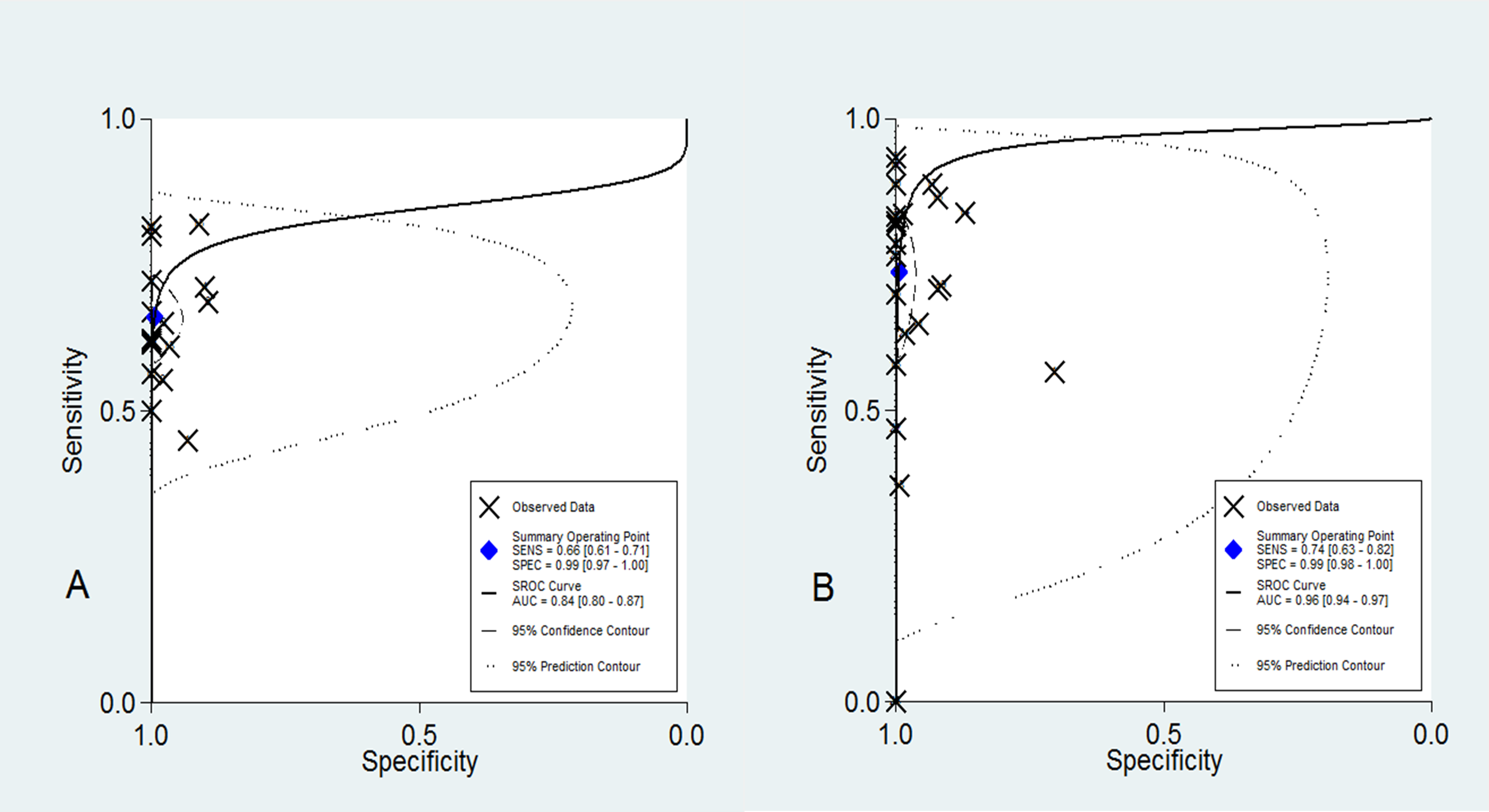

Supplement: Figure S2 — Summary ROC curve plot with sensitivity and specificity for Panbio (A) and Platelia (B). Each large X represents individual study in meta-analysis. Summary operating point is a single sensitivity/specificity point estimated by the results of studies. AUC = area under the curve. (TIF) [file pone.0094655.s002.tif]
