# Supplementary material for: A Meta-Analysis of the Diagnostic Accuracy of Two Commercial NS1 Antigen ELISA Tests for Early Dengue Virus Detection
Source: PLoS One. 2014 Apr 11;9(4):e94655. doi: 10.1371/journal.pone.0094655 (PMC3984211; doi:10.1371/journal.pone.0094655)
Supplement: Table S3 — Univariate meta-regression analyses of the sensitivity and specificity. (DOCX) [file pone.0094655.s005.docx]

**Table S3 Univariate meta-regression analyses of the sensitivity and specificity**

| **Test** | **Parameter** | **Category** | **nstudies** | **Sensitivity [95% CI]** | **p1** | **Specificity [95% CI]** | **p2** |
| --- | --- | --- | --- | --- | --- | --- | --- |
| **Panbio®** |  |  |  |  |  |  |  |
|  | Study size greater than 100? | Yes | 14 | 0.67 [0.62 - 0.72] | 0.95 | 0.99 [0.98 - 1.00] | 0.00*** |
|  |  | No | 2 | 0.57 [0.38 - 0.76] | - | 1.00 [1.00 - 1.00] | - |
|  | Origin of samples, It’s coming from Asia? | Yes | 6 | 0.59 [0.50 - 0.67] | 0.00*** | 1.00 [0.99 - 1.00] | 0.00*** |
|  |  | No | 9 | 0.70 [0.64 - 0.76] | - | 0.99 [0.98 - 1.00] | - |
|  | Samples collected at ≤ 6 days? | Yes | 13 | 0.64 [0.58 - 0.70] | 0.00*** | 0.99 [0.98 - 1.00] | 0.03* |
|  |  | No | 3 | 0.73 [0.64 - 0.83] | - | 1.00 [0.99 - 1.00] | - |
|  | Reported secondary infection? | Yes | 5 | 0.74 [0.67 - 0.81] | 0.28 | 1.00 [1.00 - 1.00] | 0.00*** |
|  |  | No | 11 | 0.62 [0.56 - 0.68] | - | 0.97 [0.94 - 1.00] | - |
|  | Study conducted during an outbreak? | Yes | 8 | 0.68 [0.61 - 0.75] | 0.13 | 1.00 [0.99 - 1.00] | 0.01** |
|  |  | No | 8 | 0.63 [0.55 - 0.71] | - | 0.98 [0.95 - 1.00] | - |
|  | Data (samples) collection was retrospective? | Yes | 5 | 0.65 [0.56 - 0.75] | 0.04* | 0.99 [0.97 - 1.00] | 0.15 |
|  |  | No | 11 | 0.66 [0.60 - 0.73] | - | 1.00 [0.99 - 1.00] | - |
| **Platelia™** |  |  |  |  |  |  |  |
|  | Study size greater than 100 ? | Yes | 20 | 0.77 [0.71 - 0.83] | 0.65 | 0.99 [0.98 - 1.00] | 0.01** |
|  |  | No | 2 | 0.73 [0.51 - 0.95] | - | 0.99 [0.94 - 1.00] | - |
|  | Origin of samples, It’s coming from Asia? | Yes | 12 | 0.73 [0.60 - 0.87] | 0.37 | 0.99 [0.98 - 1.00] | 0.04* |
|  |  | No | 10 | 0.73 [0.58 - 0.89] | - | 0.98 [0.96 - 1.00] | - |
|  | Samples collected at ≤ 6 days? | Yes | 15 | 0.75 [0.68 - 0.83] | 0.05* | 1.00 [0.99 - 1.00] | 0.04* |
|  |  | No | 5 | 0.79 [0.67 - 0.91] | - | 1.00 [1.00 - 1.00] | - |
|  | Reported secondary infection? | Yes | 7 | 0.79 [0.70 - 0.89] | 0.08 | 1.00 [0.99 - 1.00] | 0.01** |
|  |  | No | 15 | 0.76 [0.68 - 0.83] | - | 0.99 [0.98 - 1.00] | - |
|  | Study conducted during an outbreak? | Yes | 7 | 0.79 [0.70 - 0.89] | 0.07 | 1.00 [1.00 - 1.00] | 0.00*** |
|  |  | No | 15 | 0.76 [0.68 - 0.83] | - | 0.98 [0.96 - 1.00] | - |
|  | Data (samples) collection was retrospective? | Yes | 5 | 0.74 [0.60 - 0.87] | 0.03* | 0.99 [0.97 - 1.00] | 0.10 |
|  |  | No | 17 | 0.78 [0.71 - 0.84] | - | 0.99 [0.97 - 1.00] | - |

*p≤0.05; **p≤0.01; ***p≤0.00 were considered statistically significant. Meta-regression of covariates (parameter) to investigates statistical heterogeneity between subgroups analyzed.
